# The value of interleukin-27 for differentiating tuberculous pleural effusion from *Mycoplasma pneumoniae* pneumonic effusion in children

**DOI:** 10.3389/fped.2022.948862

**Published:** 2022-07-28

**Authors:** Hui Xu, Haiming Yang, Jinrong Liu, Hui Liu, Xiaolei Tang, Huimin Li, Siyu Cai, Shunying Zhao

**Affiliations:** ^1^Department of Respiratory Medicine, Beijing Children’s Hospital, Capital Medical University, National Center for Children’s Health, Beijing, China; ^2^Center for Clinical Epidemiology and Evidence-Based Medicine, Beijing Children’s Hospital, Capital Medical University, National Center for Children’s Health, Beijing, China

**Keywords:** interleukin-27 (IL-27), pleural effusion, tuberculous pleurisy, *mycoplasma*, diagnosis

## Abstract

**Objectives:**

The early diagnosis of tuberculous pleural effusion (TPE) is challenging due to the difficulty of isolating *Mycobacterium tuberculosis*, and pleural biomarkers are an optional choice. Recent studies showed that interleukin-27 (IL-27) appears to be a new accurate biomarker for TPE in adults and no related studies were reported in children. In this study, we aimed to evaluate the potential value of IL-27 in pediatric tuberculous pleurisy by detecting its levels in pleural fluid and serum.

**Methods:**

A total of 48 children with TPE and 64 children with severe *Mycoplasma pneumoniae* (MP) pneumonic effusion (SMPPE) were enrolled in this study. IL-27 concentrations were measured in serum and pleural fluid. The diagnostic yield of IL-27 was evaluated using receiver operating characteristic (ROC) curves.

**Results:**

The level of p-IL-27 in TPE showed statistically no significant difference when compared with SMPPE (*p* > 0.05). However, pleural fluid IL-27 (p-IL-27) / serum IL-27 (s-IL-27) ratio in TPE were significantly much higher than those in SMPPE (*p* < 0.05). By the analysis of the ROC curves, the diagnostic sensitivity and specificity of the p-IL-27/s-IL-27 ratio were 100% and 48.44%, respectively (cutoff value of 1.0280). The area under the ROC curve for p-IL-27/s-IL-27 was 0.7295.

**Conclusion:**

Pleural fluid IL-27 alone was not accurate in distinguishing pediatric TPE from SMPPE, which was different from the diagnostic value of IL-27 in adult studies due to the different disease spectra between children and adults. Our results implied that the p-IL-27/s-IL-27 ratio had a potential value in distinguishing TPE from SMPPE. However, the specificity of IL-27 was relatively lower and it is necessary to find a more specific marker in tuberculous pleurisy of children.

## Introduction

Globally, it is estimated that about 10 million people infected with tuberculosis (TB) in 2020 and children accounted for 11% of all TB cases **(1)**. Although pulmonary TB is the most common form of TB seen worldwide, extrapulmonary tuberculosis (EPTB) is also a serious clinical problem. The diagnosis of EPTB is often missed or made at an advanced stage of the disease when complications have already begun. A recently multicenter pediatric study from China reported that pulmonary TB, EPTB, and concurrent extrapulmonary and pulmonary TB (combined TB) accounted for 54.23, 17.76, and 28.00%, respectively (2). The second frequent form of extrapulmonary infection among pediatric inpatients in China is TPE, constituting nearly 19.15% of all EPTB and combined TB cases (2).

Tuberculous pleurisy is usually diagnosed by directly detecting *Mycobacterium tuberculosis* in pleural fluid or finding caseating granuloma in the pleural biopsy. However, classic approaches, including pleural effusion (PE) culture and Ziehl–Neelsen staining, have demonstrated inadequate sensitivity in detecting the low burden of bacteria in the patient with PE (3, 4). In contrast, the culture of *Mycobacterium tuberculosis* is also time-consuming. Both tuberculin skin test (TST) and interferon-gamma release assay (IGRA) have been proved to be unable to discriminate patients who have totally cleared *Mycobacterium tuberculosis* infection and those who have true infection in previous studies (5). Nucleic acid amplification tests have developed rapidly, such as polymerase chain reaction and Xpert MTB/RIF, which have the problem of low and variable sensitivity (6, 7). Pleural biopsy, which highly depends on the practitioner’s technical ability, is an invasive and skilled procedure. In addition, a biopsy may not be appropriate for elderly patients and children, patients with chronic diseases, and those with a bleeding tendency.

Measurement of biomarkers, such as adenosine deaminase (ADA) in the PE, has been used to discriminate TPE from malignant PE in adults (8). However, pleural fluid ADA levels can rise in a variety of situations, including parapneumonic effusion (PPE) and empyema, which are more common in children than in adults (9). Therefore, ADA had no value in discriminating TPE from other infectious PEs of children (10).

Interleukin-27 (IL-27), a member of the IL-12 family, is a heterodimeric cytokine composed of the Epstein-Barr virus-induced gene 3 (EBI3, also known as IL-27B) and the IL27-p28 subunits (11). Recent studies have showed that IL-27 appears to be a new accurate biomarker for TPE diagnosis in adults (12, 13). However, no related studies have demonstrated the role of IL-27 in pediatric tuberculous pleurisy.

Data from previous studies suggest that MP is responsible for up to 40% of community-acquired pneumonia in children aged over 5 years (14) and its highest incidence is found in the 5- to 9-year-old group (15). The occurrence of parapneumonic PE has been reported in 13–20% of MP pneumonia cases (16, 17). MP infection always develops into lower respiratory tract infections or even severe *Mycoplasma pneumoniae* pneumonia (SMPP). The PE rate was reported to be 38.9% in SMPP cases from the intensive care unit (18). The differential diagnosis of TPE and severe *Mycoplasma pneumoniae* pneumonic effusion (SMPPE) is always confused due to their shared lymphocytic predominance and similar susceptible ages. Diagnosis of MP infection is traditionally based on serology, which may require more than 2 weeks for the development of diagnostic antibodies. At present, PCR seemed to be the first choice for direct detection of MP due to its high sensitivity and specificity (19). Nevertheless, PCR tests could not distinguish between viable and non-viable organisms after antibacterial therapy (20). In addition, MP carriers usually appear with positive PCR results (21). Therefore, the clinical application of PCR was limited to some extent. Thus, the laboratory diagnosis of *Mycoplasma pneumoniae* pneumonia (MPP) is difficult. In clinical work, if drugs against MP infection do not improve in 3–5 days, the potential of SMPP would be suspected, which means the additional therapy of corticosteroids (22). However, such a strategy may lead to disseminated TB, particularly in the absence of proper TB treatment. Therefore, early differential diagnosis is extremely important for MP infection and TB infection, especially in China with a high prevalence of both diseases.

The goal of this study was to explore the potential value of IL-27 using rapid commercial IL-27 quantification kits for differentiating between TPE and SMPPE and provide recommendations for routine clinical practice.

## Materials and methods

The study protocol was approved by the Medical Ethics Committee of Beijing Children’s Hospital, National Center for Children’s Health, China. The participants’ legal guardian/next of kin provided written informed consent to participate in this study. For diagnostic investigation, 128 consecutive, non-selected patients aged 2–15 years with different causes of PEs were hospitalized in the second Department of Respiratory Medicine, Beijing Children’s Hospital Affiliated to Capital Medical University (from 2017 to 2021). All of the enrolled children had moderate to large size of pleural fluid which is available for thoracentesis. PE size was categorized on the basis of visual estimation. The moderate size was defined by occupying one-third to two-thirds of the hemithorax and the large size was defined by occupying more than two-thirds of the hemithorax.

If any one of the following criteria was met, the diagnosis of TPE was conducted: (1) positive bacterial culture of *Mycobacterium tuberculosis* (pleural tissue or PE); (2) suggested by pathological evidence (caseating granulomas or Langhans giant cells); (3) TST is positive, plus one or more of the following: PE is dominated by lymphocytes (> 50%), exudative fluid [protein > 3 g/dl or lactate dehydrogenase (LDH) > 200 U/L]; and (4) anti-TB treatment is effective (relief of clinical symptom and PE absorption), and PE caused by other reasons is excluded (23, 24). SMPPE can be diagnosed according to the typical clinical manifestations, imaging characteristics, and the fourfold increase in serum antibody titer of mycoplasma (25).

The exclusion criteria were as follows: (1) children who received steroids or anti-TB treatment before admission; (2) children with a positive TST test in the SMPPE group; (3) patients with TPE who were positive for serum antibodies against MP; and 4) patients with other chronic diseases.

Finally, forty-eight children with TPE and sixty-four children with SMPPE were enrolled. In that case, sixteen children were excluded from the study. Four patients were excluded because of receiving steroids or anti-TB therapy, six patients with TPE were excluded because of co-infected MP, four patients with SMPPE were excluded because of reported positive TST results, and two patients were excluded because of concurrent malignant neoplasm or autoimmune disease.

Pleural effusion and serum were collected from each subject within 24 h after hospitalization (before anti-tuberculous medication). First, the PE and serum specimens were centrifuged at 1,200 × *g* for 5 min and the supernatant was frozen in multiple vials at −80°C. Samples were later coded and assayed for IL-27 concentrations, which were found to be stable over the months of storage. Second, analyses of PEs for biochemical and cytological characteristics were performed in routine.

### The IL-27 measurement

The concentrations of IL-27 in PEs and sera were quantified in duplicate using an ELISA kit (Ebioscience, San Diego, CA, United States). All samples are run as a batch in the same assay. The lowest concentration of IL-27 which can be detected is 62.5 pg/ml.

### Statistical analysis

Continuous variables were expressed as mean standard deviation (SD) or medians [interquartile range (IQR)] if presented or not a normal distribution, respectively. Continuous variables were compared *via* the *t*-tests or *Wilcoxon* signed-rank tests. Receiver operating characteristic (ROC) curves were constructed and analyzed to determine the most accurate cutoff values of IL-27 in the diagnosis of tuberculous pleurisy. Data were conducted using the SAS software version 9.4.

## Results

### Characteristics of PEs

The profiles of children with PEs are illustrated in Table 1. Children with TPE or SMPPE showed an elevation of total cell counts, which were of lymphocytic predominance. The levels of ADA in pleural fluid, as well as LDH, were significantly increased in both TPE group and SMPPE group. The variables of age, total cell counts, pleural fluid lymphocytes (%), pleural fluid protein, pleural fluid glucose, pleural fluid ADA, and pleural fluid LDH were found to be statistically significant.

**TABLE 1:** Characteristics of patients with TPE and SMPPE.

	TPE (*n* = 48)	SMPPE (*n* = 64)
Age (years)	11.500 (8.090,13.170)*	7.335 (5.580,8.395)
Total cell counts (× 10^6^)	2,002.500 (902.250,3,563.000)*	365.000 (96.250,1,473.750)
Lymphocytes(%)	85.00 (76.250,90.000)*	75.000 (63.000,83.750)
Protein(g/L)	50.650 (48.630,53.600)*	40.500 (37.030,42.700)
Glucose(mmol/L)	4.690 (3.046,5.601)*	6.805 (5.941,8.765)
ADA(U/L)	56.600 (54.700,72.550)*	46.900 (43.700,67.100)
LDH(U/L)	568.500 (407.000,811.750)*	1,413.000 (1,068.250,2,858.000)

*P < 0.05, TPE compared with SMPPE, determined by the Wilcoxon signed-rank test.

### Concentration and diagnostic value of IL-27

Table 2 indicates that the levels of pleural effusion IL-27 (p-IL-27) in TPE were notably higher than in the corresponding sera (*P* < 0.05). There were no statistically significant differences in the concentrations of IL-27 between the PE and the corresponding sera in SMPPE (*P* > 0.05). The concentrations of p-IL-27 showed no statistical difference between SMPPE and TPE (*P* > 0.05). The concentrations of serum IL-27 (s-IL-27) in SMPPE were notably higher than those in TPE (*P* < 0.05). However, the difference emerging from the p-IL-27/s-IL-27 ratio was statistically higher in TPE than in SMPPE (*P* < 0.05).

**TABLE 2:** IL-27 levels in pleural cavity and sera.

	TPE (*n* = 48)	SMPPE (*n* = 64)
p-IL-27 (pg/ml)	89.922(84.835,97.917)*	92.801(86.596,110.362)
s-IL-27 (pg/ml)	76.877(74.076,80.510)	90.157(80.578,103.146)^#^
p-IL-27/s-IL-27 ratio	1.188(1.099,1.280)^#^	1.034(0.903,1.149)

*P < 0.05, compared with the corresponding compartments in sera, determined by the Wilcoxon signed-rank test;^#^*P* < 0.05, TPE compared with SMPPE, determined by the Wilcoxon signed-rank test.

**FIGURE 1:**
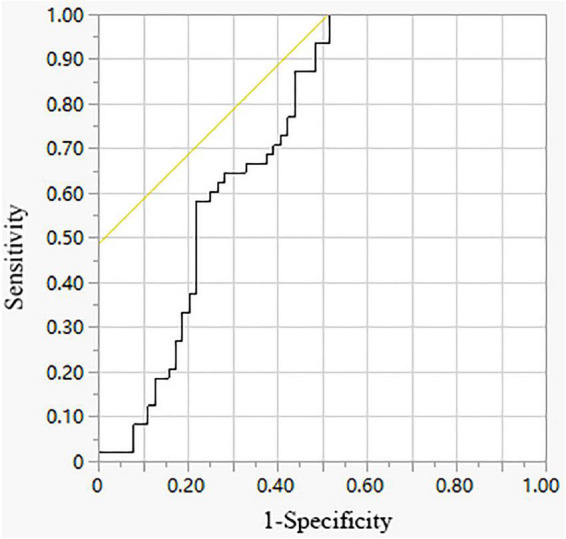
ROC curve of p-IL-27/s-IL-27 for differential diagnosing TPE from SMPPE.

The diagnostic accuracy of p-IL-27/s-IL-27 was evaluated by the ROC curve. As shown in Table 3 and Figure 1, the area under the curve (AUC) for p-IL-27/s-IL-27 when used to differentiate TPE from SMPPE was 0.7295. With a cutoff value of 1.0280, sensitivity was 100%, and specificity was 48.44%.

**TABLE 3:** Diagnostic value of p-IL-27/s-IL-27 in differentiating TPEs from SMPPPEs.

	Cut-off	AUC	Sensitivity(%)	Specificity(%)	True- positive (N)	True- negative (N)	False- positive (N)	False- negative (N)
p-IL-27/s-IL-27	1.0280	0.7295	100	48.44	48	31	33	0

AUC, area under the curve.

## Discussion

Parapneumonic effusion and pleural empyema, which are mainly associated with bacterial infections, represent the main cause of pleural infections. For years, *Streptococcus pneumoniae* has been invariably found the most common etiologic factor of community-acquired pneumonia. Cases of pneumococcal PEs decreased sharply among 2- to 5-year-old children following the introduction of the pneumococcal conjugate vaccine (26). While the diagnosis of PPE and empyema induced by bacteria is relatively simple (e.g., acute febrile disease, or pus withdrawn during thoracentesis), *Mycoplasma pneumoniae* pneumonic effusion (MPPE) frequently presents as a lymphocytic exudate with elevated ADA levels mimicking TPE. The misdiagnosis between TPE and MPPE has also been reported even in adults (27, 28). In pediatric patients in China, with a high incidence of both TB and MP infection, the differentiation between TPE and SMPPE is of great importance.

All the variables from Table 1 were found to be statistically significant. In our previous study, we have already performed simple and multivariate logistic regression analyses to identify characteristics for discrimination between TPEs and SMPPEs. In addition, the results discovered that blood neutrophils and serum LDH were significant independent factors to discriminate between TPEs and SMPPEs (29). The results from this study proved again that the pleural ADA level increased in both TPE and SMPPE groups, which is consistent with the reported literature (30). Results have shown that there were no statistically significant differences in the concentrations of p-IL-27 in TPE compared with SMPPE, which indicates that the p-IL-27 level is of no value in differentiating between TPE and MPPE. However, the difference emerging from the p-IL-27/s-IL-27 ratio had a potential value in differentiating TPE from SMPPE. We also validated the cutoff values of the p-IL-27/s-IL-27 ratio for TPE diagnosis with considerable sensitivity and specificity. To the best of our knowledge, there are no similar results reported in the existing studies. Consistent with the results of previous study (12), our results proved that the level of s-IL-27 was much lower than in corresponding PE of TPE, which confirmed that the increased IL-27 level in pleural fluid with TPE was caused by local pleural production rather than passive diffusion from plasma to the pleural cavity. In contrast, the results showed that no statistical differences were found in the level of IL-27 between PE and corresponding sera in SMPPE. However, the s-IL-27 level of SMPPE was significantly higher than that in TPE. The cell-mediated immune response is proved to be significant during the pathogenesis of MPP. Recent research also revealed that inflammatory cytokines play an important role in immunopathogenesis after MP infection (31, 32). Although no relevant studies have been conducted to investigate the involvement of IL-27 in the pathophysiology of MPP, we speculate that IL-27 may participate in the pathogenesis of MPP by a passive diffusion from plasma to the pleural cavity. Elevated serum IL-10 could be used for differentiating patients with refractory MPP from those with general MPP in school-aged children (33, 34). IL-10, known as one of the anti-inflammatory cytokines, has a central role in infection by limiting the immune response to pathogens, thereby preventing damage to the host (35). IL-27 was initially regarded as a pro-inflammatory cytokine that had an ability to induce T helper 1 cell immunity. However, it was later discovered that IL-27 was also an anti-inflammatory cytokine with upregulation of T-bet and IL-10 which could inhibit the differentiation of Th2 cells (36). Three recent reports demonstrated that IL-27 promotes IL-10 production by CD4 + T cells (37–39). Therefore, the inflammatory effect of IL-27 in MPP might be associated with increased production of IL-10. Using the ROC curve, the AUC is 0.7295 for the p-IL-27/s-IL-27 ratio to diagnose TPE. Based on the highest diagnostic accuracy with the highest sensitivity, the cutoff value for p-IL-27/s-IL-27 was set to be 1.0280. Using this cutoff value, the sensitivity and specificity of the p-IL-27/s-IL-27 ratio for TPE were 100% and 48.44%, respectively. The specificity of cutoff value is not ideal, which may be due to the limited sample size of this study. The patient with confirmed TB needs to be referred to specialized hospitals in China for further treatment and management. Therefore, TB is often misdiagnosed because of the lack of clinician awareness and inadequate diagnostic capacity by doctors from general hospitals. The specificity of p-IL-27/s-IL-27 was low but with high sensitivity. It might be reasonable to use the index with higher sensitivity to reduce the missed diagnoses of TPE, which is in line with the clinical needs in China.

The concentration of IL-27 in PEs was found to be increased in tuberculous pleurisy and several adult studies have investigated the diagnostic value of IL-27 for TPEs, but the reported results remain controversial. A study from 2014 showed that the efficiency of IL-27 in TPE was lower than ADA and ADA-2. However, ADA&IL-27 and ADA-2&IL-27 can improve the diagnostic sensitivity of ADA and ADA-2, respectively. Therefore, IL-27 may be useful in PE with high suspicion of TPE and low ADA level (40). Wu et al. demonstrated that compared with ADA and interferon gamma (IFN-γ), IL-27 appeared to be more accurate for distinguishing tuberculous from non-TPEs, especially from malignant PE (41). The recent meta-analysis provided more details to prove that pleural IL-27 being a valuable biomarker for the diagnosis of TPE and its diagnostic efficiency is significantly better than that of ADA or IFN-γ (13). Results in our study were not fully consistent with the previous research on adults, in which the control groups are mainly malignant patients. Malignant PEs exhibit a high incidence among adults. However, infectious PEs are more common than malignant PE in children. MPPE in this study was chosen as the control group instead of malignant PE based on different disease spectra in children. From this study, we found an elevated p-IL-27 in both TPE and MPPE which demonstrated that p-IL-27 alone was not proved effective in discriminating TPE from MPPE.

For the first time, our study has shown the diagnostic role of IL-27 in pediatric tuberculous pleurisy. Although our study demonstrated the role of IL-27 in the differential diagnosis of TPE and MPPE, it still had several limitations. First, we enrolled only children with MPPE as a control group. The current studies did not include transudates from different origins, such as heart failure and liver cirrhosis. However, transudative PEs can be distinguished from exudative PEs based on Light’s criteria in most cases. More prospective research is needed to explore the role of IL-27 in a broader group of individuals induced by a variety of etiologies. Second, the severity of the disease was not taken into consideration. Third, we did not conduct a serial analysis of IL-27 levels after anti-TB therapy. Finally, the sample size was small. The results might be limited and more external validation is needed. However, the mechanism of elevated IL-27 in TPE remains to be further explored.

In this study, we showed for the first time the value of IL-27 in discriminating pediatric tuberculous pleurisy from SMPPE. However, any biomarkers or cytokines cannot replace bacterial culture, which remains necessary for the diagnosis of TB.

## Conclusion

Our findings indicated that the p-IL-27/s-IL-27 ratio may be a possible pleural biomarker to distinguish TPE and SMPPE. However, unlike the results from the adults, the specificity of IL-27 is relatively lower, which may be due to the small sample size and different control groups based on different disease spectra. Hence, researchers should endeavor to look for more effective markers in tuberculous pleurisy of children.

## Data availability statement

The raw data supporting the conclusions of this article will be made available by the authors, without undue reservation.

## Ethics statement

The studies involving human participants were reviewed and approved by Medical Ethics Committee of Beijing Children’s Hospital, National Center for Children’s Health, China. The participants’ legal guardian/next of kin provided written informed consent to participate in this study.

## Author contributions

HX contributed to the conception and designed the study, conducted the analysis, drafted the initial manuscript, and reviewed and revised the initial manuscript. HY, JL, HL, XT, and HmL helped with study design, overviewed the study, and revised the manuscript. SC analyzed and interpreted the data. SZ conceptualized and designed the study, analyzed the data, and agreed with manuscript results and conclusions. All authors reviewed and approved the final manuscript.

## Conflict of interest

The authors declare that the research was conducted in the absence of any commercial or financial relationships that could be construed as a potential conflict of interest.

## Publisher’s note

All claims expressed in this article are solely those of the authors and do not necessarily represent those of their affiliated organizations, or those of the publisher, the editors and the reviewers. Any product that may be evaluated in this article, or claim that may be made by its manufacturer, is not guaranteed or endorsed by the publisher.

## Abbreviations

TB: Tuberculosis
TPE: Tuberculous pleural effusion
MP: *Mycoplasma pneumoniae*
SMPPE: Severe *Mycoplasma pneumoniae* pneumonic effusion
IL-27: Interleukin-27
ADA: Adenosine deaminase
IFN-γ: Interferon gamma
ROC: Receiver operator characteristic.
